# Specific Lipid Peroxidation Products in Erythrocytes and Their Relationship to the Pathogenesis of Alzheimer's Disease

**DOI:** 10.1111/jcmm.70990

**Published:** 2026-02-27

**Authors:** Lenka Martináková, Zuzana Chmátalová, Kateřina Veverová, Vanesa Jurášová, Alžběta Katonová, Martina Laczó, Jan Laczó, Martin Vyhnálek, Jakub Hort, Alice Skoumalová

**Affiliations:** ^1^ Department of Medical Chemistry and Clinical Biochemistry, Second Faculty of Medicine Charles University and Motol University Hospital Prague Czech Republic; ^2^ Memory Clinic, Department of Neurology, Second Faculty of Medicine Charles University and Motol University Hospital Prague Czech Republic

**Keywords:** alzheimer's disease, cerebrospinal fluid biomarkers, lipofuscin‐like pigments, neurodegeneration, oxidative stress

## Abstract

Alzheimer's disease (AD) is a highly complex and multifactorial disorder in which oxidative stress acts as a key amplifying mechanism in the disease progression. Lipofuscin‐like pigments (LFP), end products of lipid peroxidation, reflect oxidative damage and are capable of crossing the blood–brain barrier into the circulation. Thus, erythrocyte‐derived LFP may serve as peripheral indicators of brain‐specific processes. In this study, we aimed to assess the specificity of previously identified LFP for AD pathology. We analysed erythrocyte‐derived LFP in individuals with biomarker‐verified AD pathology (*n* = 40) and non‐AD cognitive disorders (*n* = 21) across the prodromal and dementia stages, and in cognitively unimpaired individuals (*n* = 19). AD individuals showed significantly higher LFP levels compared with both healthy controls (*p* < 0.003) and the non‐AD group (*p* ≤ 0.001). Furthermore, LFP levels correlated with established AD biomarkers in CSF, including amyloid‐beta (*r* > −0.566) and phosphorylated tau 181 (*r* < 0.477). Our findings suggest that LFP may serve as potential blood‐based biochemical markers reflecting oxidative stress–related processes associated with AD pathology.

## 1 Introduction

Alzheimer's disease (AD) is a severe neurodegenerative disorder considered to be the most common cause of dementia [1]. The terminal stage of dementia, characterised by the loss of self‐sufficiency, is typically preceded by a prodromal phase known as mild cognitive impairment (MCI), during which patients retain intact activities of daily living [2, 3]. According to the widely accepted amyloid cascade hypothesis, the pathogenesis of AD is initiated by the accumulation of amyloid‐beta (Aβ) peptides, which subsequently trigger a sequence of pathological events, including hyperphosphorylation of tau protein. These molecular alterations are accompanied by neuroinflammatory responses and oxidative stress (OS), which serve as major pathogenic cofactors contributing to the progression of neuronal damage and cognitive decline [4, 5, 6]. Current evidence suggests that Aβ aggregation both arises under conditions of increased OS and actively enhances oxidative damage, establishing a pathophysiological feedback loop that accelerates neurodegeneration. Although it remains unresolved whether OS precedes or follows Aβ and tau pathology, its role as a central mediator of disease progression is well established [5, 6, 7].

The brain tissue is susceptible to oxidative damage due to the rich content of polyunsaturated fatty acids, which are prone to lipid peroxidation, resulting in the production of highly reactive compounds such as malondialdehyde and 4‐hydroxynonenal [8, 9]. These lipid peroxidation products further react with brain phospholipids and proteins, forming complex lipid peroxidation end products called lipofuscin‐like pigments (LFP), which exhibit characteristic natural fluorescence [10]. Lipid peroxidation intermediates generated in brain tissue can diffuse across the blood–brain barrier into the bloodstream, where they interact with phospholipids and proteins in erythrocyte membranes, forming detectable LFP. Given the specific lipid composition of neuronal membranes, erythrocyte‐derived LFP exhibits unique biochemical and fluorescence profiles reflective of brain lipid peroxidation [11].

Considering that LFP specifically reflects the OS associated with AD, they represent promising candidates for biomarkers to elucidate underlying pathophysiological mechanisms and to monitor the efficacy of disease‐modifying therapies. In our previous research, we identified significantly elevated levels of LFP in the blood of individuals with AD. Notably, LFP levels were increased in both erythrocytes and plasma across all symptomatic stages of AD (including MCI and dementia) compared to healthy controls [12, 13]. The previous studies were limited to clinically diagnosed AD cases, without biomarker confirmation. Additionally, LFP levels were not compared with those in other dementing conditions, leaving it unclear whether the observed LFP accumulation is specific to AD pathology.

The present study builds on our previous results [13], by further exploring and validating the relationship between erythrocyte LFP levels and AD pathology using biomarker‐defined study groups. To test the specificity of LFP in AD, we included a comparison with LFP from other non‐AD neurodegenerative diseases. The non‐AD group consisted of participants with Lewy body disease (LBD) and frontotemporal lobar degeneration (FTLD). These neurodegenerative diseases were chosen because they are characterised by the accumulation of pathological proteins in the brain and are accompanied by OS [14, 15, 16, 17]. The first aim of this study was to compare the LFP levels in biomarker‐defined individuals across prodromal and dementia stages of AD and to validate fluorescence maxima previously identified by our group [13]. The second aim of this study was to evaluate the specificity of LFP for AD by comparing its levels with those observed in individuals with non‐AD neurodegenerative disorders. The third aim was to investigate the relationship between LFP levels and AD‐related pathological changes, as reflected by the level of biomarker abnormality. Our results confirmed a significantly higher LFP accumulation in erythrocytes of individuals with AD, while such increases were not present in either cognitively unimpaired individuals or individuals with non‐AD neurodegenerative conditions. Another interesting finding was that the amount of LFP in several fluorescence maxima correlated with AD pathology biomarkers. These findings suggest that LFP may serve as a promising blood‐based biochemical marker of OS‐related pathophysiological processes in AD.

## 2 Materials and Methods

### 2.1 Participants

We analysed blood samples from 80 subjects recruited by the Department of Neurology, Motol University Hospital in Prague, Czech Republic. Study participants were selected from the Czech Brain Aging Study, a longitudinal study on aging and cognitive impairment [18]. The study cohort included 26 participants diagnosed with MCI due to AD (AD‐MCI), 14 with AD dementia (ADD) and 19 cognitively unimpaired individuals (CU) [19, 20]. We also included other non‐AD neurodegenerative diseases (LBD and FTLD) that were divided into MCI due to LBD/FTLD (non‐AD MCI group) with 10 participants and LBD/FTLD dementia stage (non‐ADD group) with 11 participants [21, 22]. The detailed characteristics of study groups are described in (Table 1).

**TABLE 1:** Demographic and clinical characteristics of the study groups.

	AD‐MCI	ADD	Non‐AD MCI	Non‐ADD	CU
Cohort size (women %)	*n* = 26 (46.15%)	*n* = 14 (50.00%)	*n* = 10 (30.00%)	*n* = 11 (45.45%)	*n* = 19 (73.68%)
MMSE, mean ± SD (range)	26 ± 2.14 (22–30)	18 ± 4.43 (10–23)	27 ± 3.12 (22–30)	20 ± 3.99 (14–25)	29 ± 0.83 (27–30)
Age, mean ± SD (range)	73 ± 6.92 (54–83)	71 ± 8.00 (56–83)	69 ± 8.56 (52–82)	68 ± 9.32 (54–82)	70 ± 8.94 (55–92)
CSF Aβ42, mean ± SD (range)	445.46 ± 68.22 (334.70–554.9)	423.56 ± 60.76 (300.90–523.00)	1309.73 ± 589.45 (695.20–2261.8)	1016.64 ± 333.43 (578.90–1679.40)	
CSF p‐tau181, mean ± SD (range)	123.07 ± 83.77 (34.10–358.80)	101.57 ± 74.46 (36.90–312.10)	60.54 ± 34.48 (17.40–122.90)	62.73 ± 46.81 (15.50–164.50)	
CSF t‐tau, mean ± SD (range)	493.47 ± 307.32 (101.60–1204.50)	565.69 ± 238.83 (174.9–902.60)	271.13 ± 136.68 (92.80–480.90)	386.77 ± 217.95 (211.90–827.00)	

*Abbreviations:* AD‐MCI, MCI due to AD; ADD, AD at dementia stage; CSF Aβ42, amyloid beta 42 tested in CSF; CSF p‐tau181, phosphorylated tau 181 tested in CSF; CSF t‐tau, total tau tested in CSF; non‐AD MCI, MCI due to non‐AD; non‐ADD, non‐AD at dementia stage; CU, cognitively unimpaired individuals; MMSE, Mini‐Mental State Examination (score range 0–30); SD, standard deviation.

All participants involved in the study signed informed consent approved by the Ethics Committee of Motol University Hospital, and the investigated biological samples were coded to ensure participant anonymity. All participants underwent comprehensive neuropsychological and neurological assessments and brain magnetic resonance imaging. AD aetiology was confirmed either by cerebrospinal fluid (CSF) biomarkers amyloid beta 42 (Aβ42), phosphorylated tau protein 181 (p‐tau181) and total tau (t‐tau) or by Aβ detection via positron emission tomography (PET). Participants with severe brain vascular burden (Fazekas score > 2 on brain magnetic resonance imaging), other neurological and psychiatric diseases (e.g., multiple sclerosis, epilepsy, traumatic brain injury, stroke, psychotic or schizoaffective disorders, major depressive disorder, anxiety disorders and obsessive compulsive disorder), systemic diseases that can cause cognitive impairment (e.g., renal, hepatic and cardiac failure, decompensated diabetes and oncological diseases), and a history of alcohol or drug abuse were not included in the study.

### 2.2 Blood Sampling, LFP Extraction and LFP Fluorescence Analysis

Venous blood was collected into K_3_EDTA‐coated tubes and centrifuged within 20 min after sampling at 4000 *g* for 5 min. The separated plasma was removed, and the sediment (erythrocytes) was stored at −80°C until analysis.

The modified technique described by Goldstein and McDonagh was used to extract LFP from erythrocyte suspensions [12, 13, 23]. Organic extracts containing LFP were stored at −20°C until fluorimetric analysis. All analyses were performed on erythrocyte extracts prepared in tetraplicates for each subject to minimise technical variability.

The fluorescence characteristics of LFP extracts were analysed on AMINCO‐Bowman Series 2 spectrofluorometer. At first, three‐dimensional (3D) analysis was performed for excitation wavelength in the range of 250–400 nm and emission in the range of 300–500 nm. Then the synchronous (SYN) spectra were measured in the wavelength range of 250–500 nm for emission with two fixed differences for excitation, 25 nm (SYN25) and 50 nm (SYN50). For quantitative analyses, the fluorescence maxima identified in the previous study [13], were used: 290/350 nm, 333/358 nm, 285/335 nm, 310/360 nm and 350/400 nm (excitation/emission). The fluorescence determined at wavelength 260/480 nm (excitation/emission) was used as a reference.

### 2.3 CSF Sampling, Analysis of CSF Biomarkers and Amyloid PET Imaging

CSF samples were obtained by lumbar puncture in the supine position, collected in 8‐mL polypropylene tubes, combined, gently mixed and centrifuged at 1700 *g* at 20°C for 5 min within 30 min of collection. CSF was aliquoted in polypropylene tubes of 0.5 mL and stored at −80°C until analysis. Stored CSF samples were thawed and vortexed before biomarker analysis. CSF collection, processing and archiving were performed in accordance with European recommendations [24]. CSF biomarkers (Aβ42, p‐tau181 and t‐tau) were analysed using commercial ELISA kits (Euroimmun). The following cut‐offs for negative results were provided by Euroimmun: Aβ42 > 550 pg/mL and p‐tau181 < 61 pg/mL.

The PET images were acquired using a Biograph 40 TrueV HD PET/CT scanner (Siemens Healthineers AG). The participants received a single intravenous dose of flutemetamol (18F; Vizamyl, GE Healthcare). Noncontrast low‐dose CT brain images were acquired for attenuation correction prior to the PET scans. A PET list‐mode acquisition was performed in two phases: early (perfusion) and late (β‐amyloid). The early‐phase images were acquired at the time of flutemetamol (18F) administration for 8 min and the late‐phase images were acquired 90 min after flutemetamol (18F) administration for a total of 10 min. Flutemetamol (18F) PET images were visually read (as positive or negative) by a certified nuclear medicine specialist using the GM‐EDGE method [25].

### 2.4 AT(N) Biomarker System

The AT(N) biomarker system was used to investigate the relationship between the erythrocyte LFP amount and AD pathology. In this classification, subjects are categorised based on CSF biomarkers into three domains (‘A’ as pathological Aβ, ‘T’ as pathological tau, ‘N’ as neurodegeneration), which reflects different stages and types of pathology [26, 27, 28, 29]. All participants undergoing CSF analysis (*n* = 44) were classified according to the AT(N) criteria framework. To define the AT(N) status, we used CSF Aβ42 as ‘A’, CSF p‐tau181 as ‘T’ and CSF t‐tau as ‘N’ [26]. These biomarkers were dichotomised as normal (−) or abnormal (+), and the patients were divided into groups, which were stratified based on biomarker levels (Table 2).

**TABLE 2:** AT(N) classification of the study groups.

AT(N) profile	Cohort size	Clinical cohort (characteristics)	
A+T−N−	*n* = 5	AD individuals—Alzheimer's continuum	AD pathological change
A+T+N−	*n* = 5		AD pathology
A+T+N+	*n* = 18		AD pathology
A−T+N−	*n* = 4	Non‐AD individuals with pathological changes	
A−T+N+	*n* = 4		
A−T−N+	*n* = 1		
A−T−N−	*n* = 7	Non‐AD individuals with normal biomarkers	

Abbreviations: AD, Alzheimer's disease; CSF Aβ42, amyloid beta 42 tested in CSF; CSF p‐tau181, phosphorylated tau 181 tested in CSF; t‐tau, total tau tested in CSF. *Notes*: ‘+’ indicates an abnormal biomarker, ‘−’ indicates a normal biomarker, ‘A’ = CSF Aβ42 levels; ‘T’ = CSF p‐tau181 levels; ‘N’ = CSF t‐tau levels.

### 2.5 Statistical Analysis

All statistical analyses and outputs were performed using the StatView software. Differences between the groups were assessed using an ANOVA, followed by Bonferroni/Dunn post hoc tests. Only results with a statistical power of analysis greater than 0.900 are reported. The threshold for statistical significance was set at 0.05, and *p* ≤ 0.05 (*), 0.01 (**), 0.001 (***) were considered statistically significant. To assess the influence of age and sex on quantitative analyses of LFP levels across the groups, ANCOVA was performed. Pearson's correlation coefficient was used to examine the relationship between LFP levels and biomarker levels in CSF. Multiple linear regression analysis was applied to determine whether age and sex had a moderating effect on this relationship.

## 3 Results

### 3.1 Fluorescence Analyses of LFP in Erythrocyte Extracts

**FIGURE 1:**
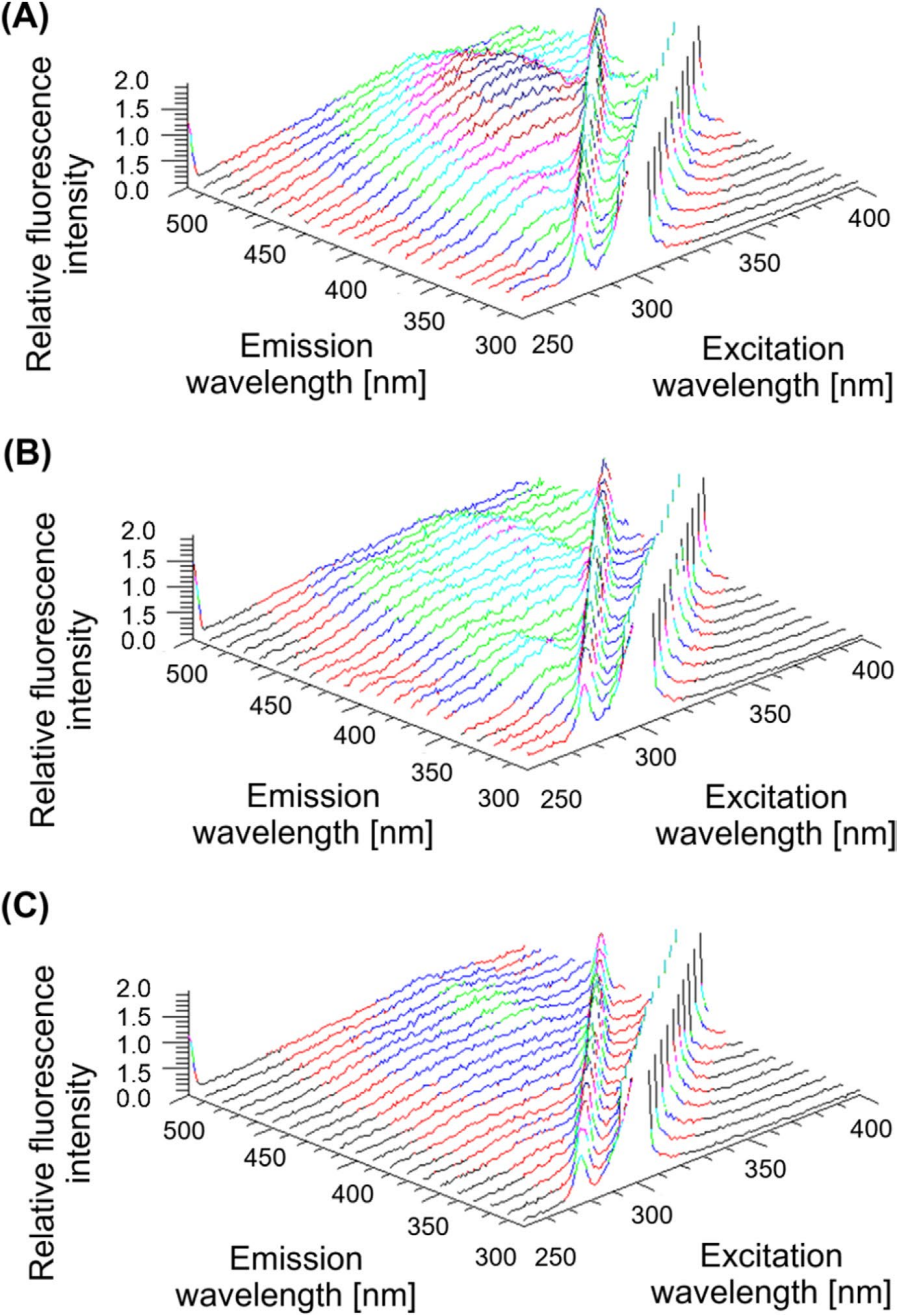
Three‐dimensional (3D) fluorescence spectra of erythrocyte‐derived lipid peroxidation products in individuals with dementia stage of Alzheimer's disease, ADD group (A), with prodromal stage of Alzheimer's disease, AD‐MCI group (B) and cognitively unimpaired individuals, CU group (C). Fluorescence intensity is visualised using a colour scale.

The LFP extracts were analysed by fluorescence spectroscopy. In accordance with our previous research [13], we confirmed qualitative and quantitative differences between AD pathology (AD‐MCI and ADD) and CU. At first, 3D fluorescence spectra were performed. Higher fluorescence intensity in the 3D spectra was detected in AD participants (AD‐MCI and ADD) compared to the CU group, with the most pronounced differences in the excitation wavelength range of 290–360 nm and the emission range of 350–460 nm. No significant differences between AD‐MCI and ADD were detected. An example of the 3D fluorescence spectra is presented in (Figure 1).

**FIGURE 2:**
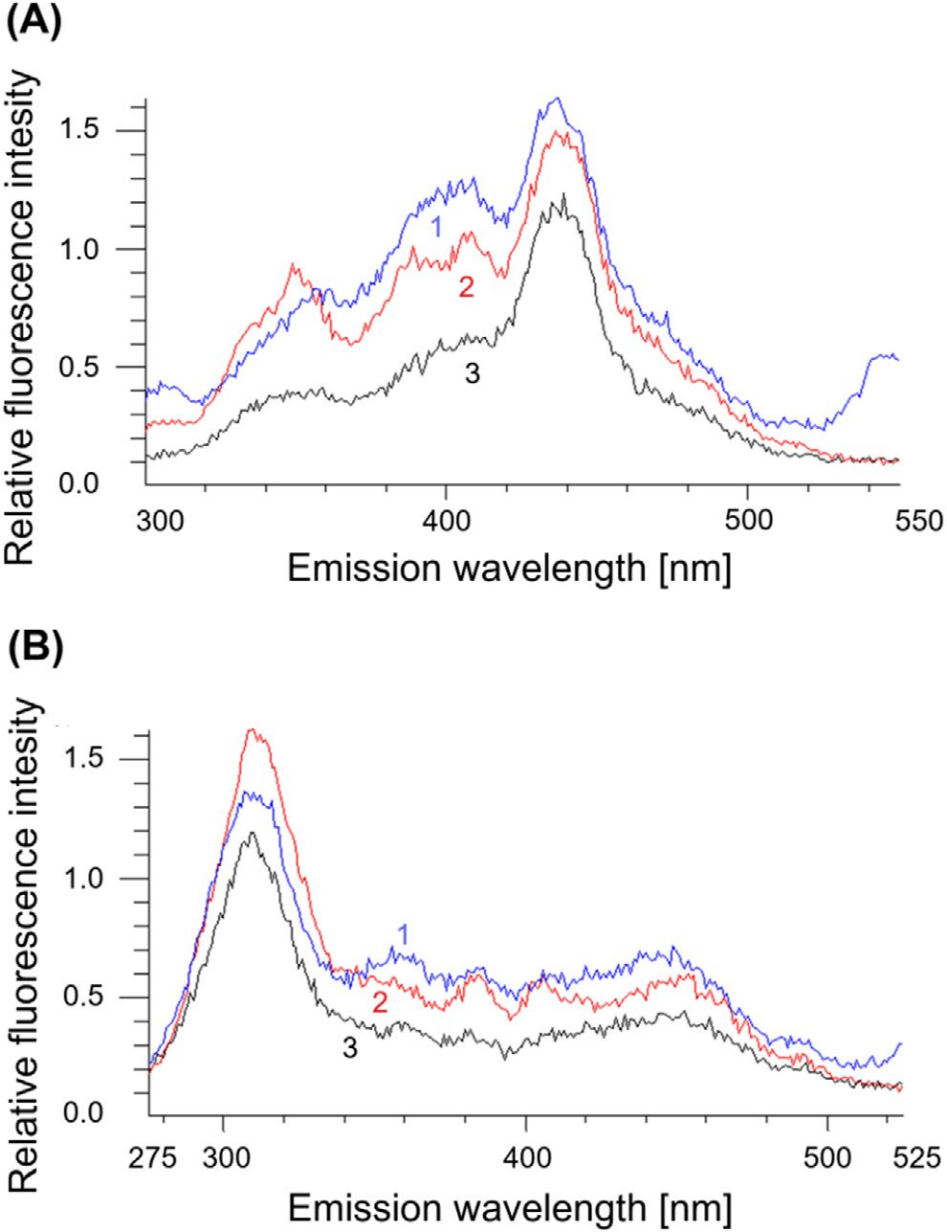
Synchronous fluorescence spectra, SYN50 (A), SYN25 (B) of erythrocyte‐derived lipid peroxidation products in individuals with dementia stage of Alzheimer's disease, ADD group (curve 1), with prodromal stage of Alzheimer's disease, AD‐MCI group (curve 2) and cognitively unimpaired individuals, CU group (curve 3).

To obtain more detailed fluorescence characteristics, we performed analysis using synchronous fluorescence spectra (SYN50, SYN25). In the SYN50 spectra, we identified qualitative and quantitative differences between AD‐MCI and ADD versus CU. Higher fluorescence intensity was detected in AD participants (both AD‐MCI and ADD) compared to CU, particularly at emission wavelengths around 350 nm and 400 nm. No significant differences were observed between AD‐MCI and ADD. In the SYN25 spectra, we also observed quantitative and qualitative differences. Although fluorescence intensities were elevated in AD individuals (AD‐MCI and ADD) compared to CU, the differences were not significant. An example of the SYN spectra is shown in (Figure 2).

### 3.2 Quantitative Analysis of LFP in Erythrocyte Extracts

**FIGURE 3:**
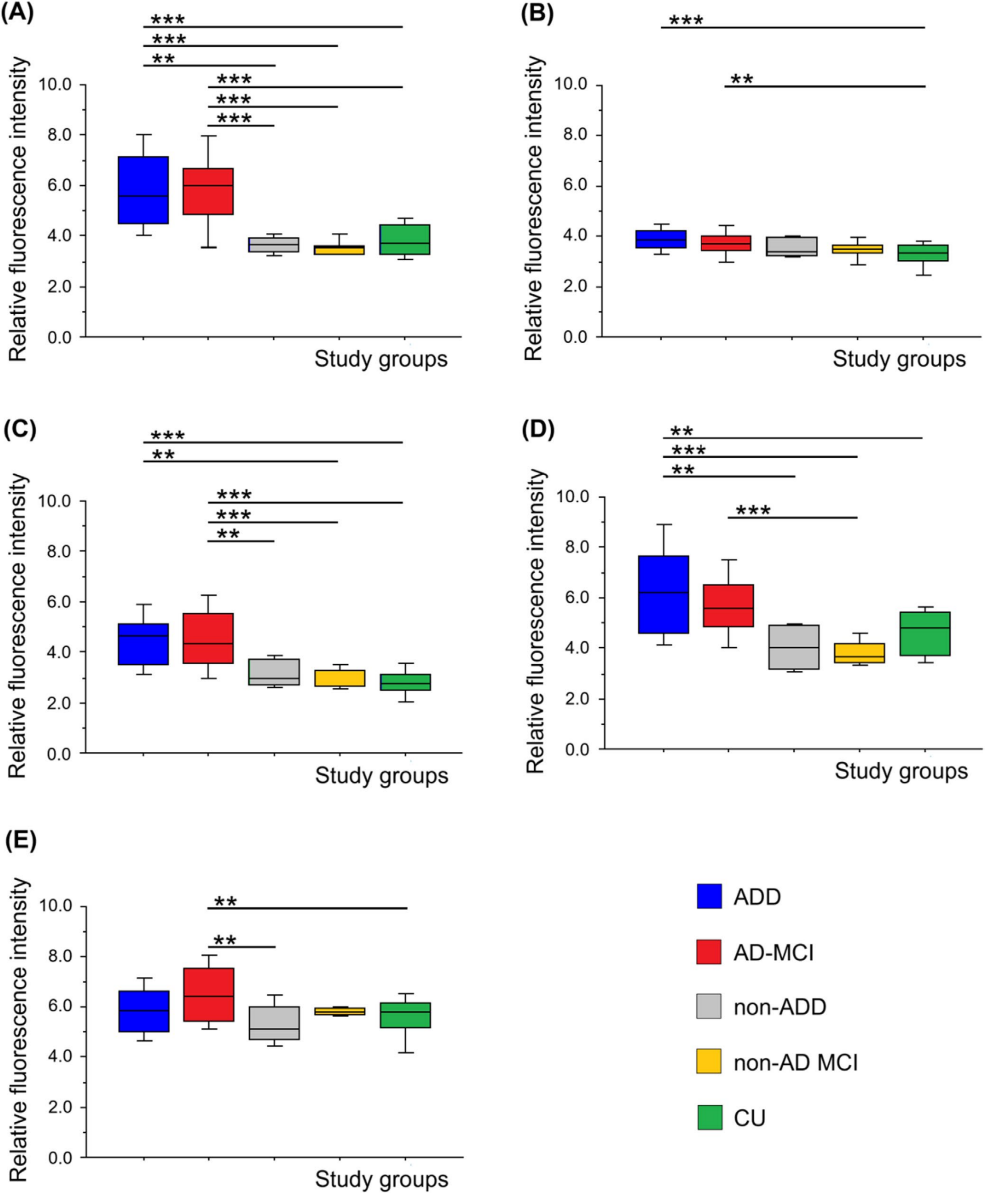
Quantitative analysis of relative fluorescence intensity of erythrocyte‐derived lipid peroxidation products across study groups (AD‐MCI, ADD, non‐AD MCI, non‐ADD and CU) at five specific fluorescence maxima, excitation/emission: 290/350 nm (A), 333/358 nm (B), 285/335 nm (C), 310/360 nm (D), 350/400 nm (E). Data were statistically analysed by Analysis of Variance (ANOVA) followed by Bonferroni/Dunn post hoc test. **, *p* ≤ 0.01, ***, *p* ≤ 0.001. Data are presented as box plots generated using StatView software.

The significant fluorescence maxima identified in our previous study [13] in 3D and SYN fluorescence spectra, were further used for quantitative analyses to confirm whether blood LFP was increased in individuals with AD pathology compared to CU. Moreover, the comparison of LFP in AD versus non‐AD aetiology in MCI, as well as in the dementia stage, was performed. Hereby, we wanted to investigate whether elevated LFP levels are specific to AD aetiology. The effects of age and sex on LFP values at fluorescence maxima across study groups were not statistically significant, as determined by ANCOVA (*p* > 0.05). Although the statistical power was below the recommended threshold (< 0.80), we assume that neither age nor sex had a substantial impact on LFP levels. Therefore, the results are presented and interpreted based on ANOVA. Statistically significant differences in fluorescence intensity were observed between the participants with AD pathology (AD‐MCI and ADD) and CU (Figure 3). The results showed significantly higher fluorescence intensity in AD‐MCI compared to CU at three fluorescence maxima, 290/350 nm (*p* < 0.001), 333/358 nm (*p* = 0.002) and 285/335 nm (*p* < 0.001). Significantly higher fluorescence intensity was also observed in ADD compared to CU at four fluorescence maxima, 290/350 nm (*p* < 0.001), 333/358 nm (*p* < 0.001), 285/335 nm (*p* < 0.001) and 310/360 nm (*p* = 0.003). Fluorescence intensities at all analysed maxima were comparable between the AD‐MCI and ADD groups.

Furthermore, we wanted to know if the identified specific fluorescence maxima were significantly higher in AD versus non‐AD pathology (Figure 3). The results showed significantly higher fluorescence intensity in AD‐MCI compared to non‐AD MCI at three fluorescence maxima, 290/350 nm (*p* < 0.001), 285/335 nm (*p* < 0.001) and 310/360 nm (*p* = 0.001), and in AD‐MCI compared to non‐ADD at two fluorescence maxima, 290/350 nm (*p* = 0.001) and 285/335 nm (*p* = 0.001). The significantly higher fluorescence intensity was also observed in ADD compared to non‐AD MCI at the same three fluorescence maxima, 290/350 nm (*p* < 0.001), 285/335 nm (*p* = 0.001) and 310/360 nm (*p* < 0.001), and in ADD compared to non‐ADD at two fluorescence maxima, 290/350 nm (*p* = 0.001) and 310/360 nm (*p* = 0.001). The significantly higher fluorescence intensity was also observed in AD‐MCI compared to CU and to non‐ADD at 350/400 nm fluorescence maximum, although with a lower statistical power (0.872).

**FIGURE 4:**
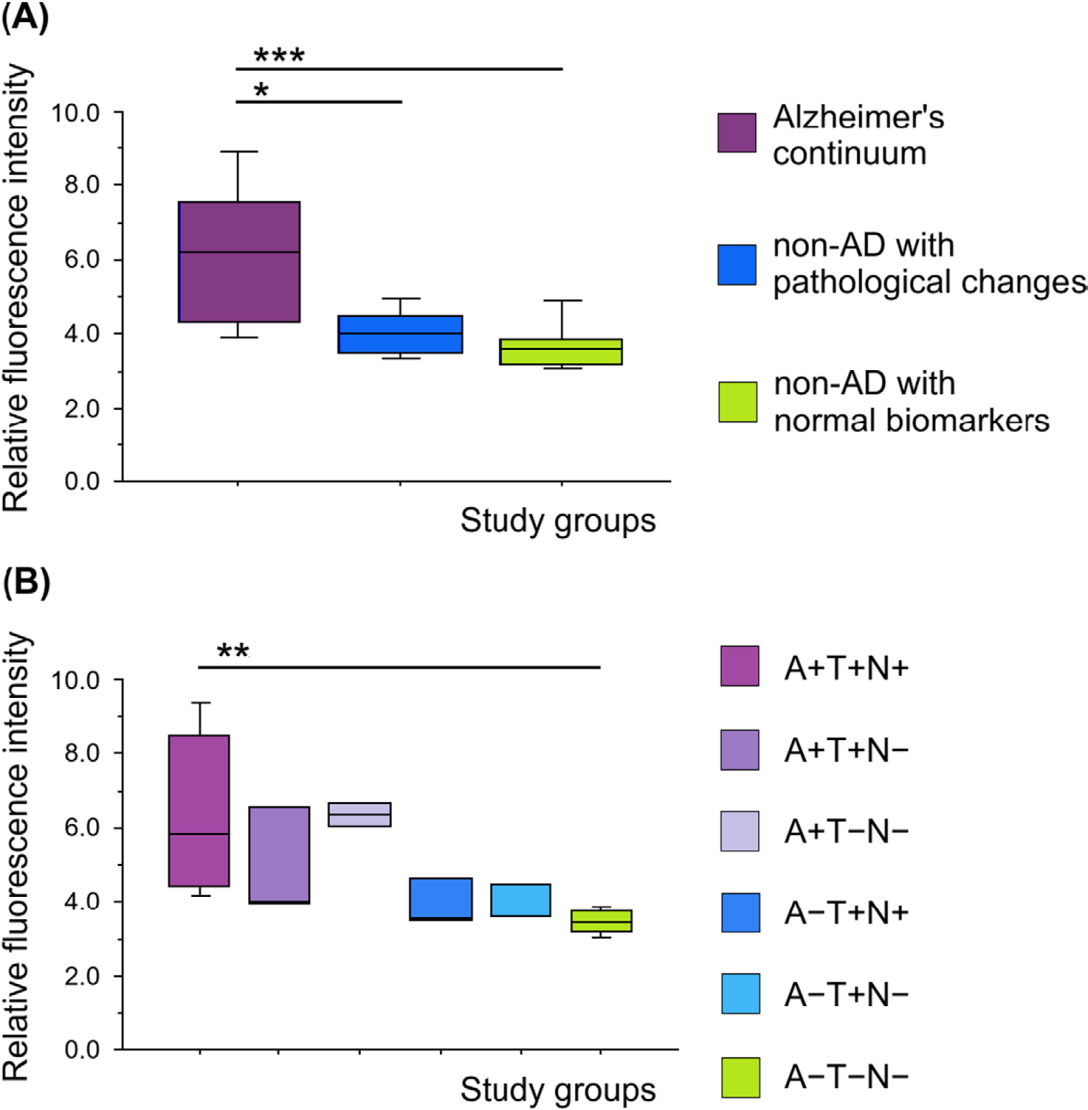
Quantitative analysis of relative fluorescence intensity (at fluorescence maximum 310/360 nm) of erythrocyte‐derived lipid peroxidation products in diagnostic groups classified according to the AT(N) framework. Analysis of three main groups: Alzheimer's continuum group (A+T−N−, A+T+N−, A+T+N+), group of non‐AD individuals with pathological changes (A−T+N−, A−T+N+, A−T−N+) and group of non‐AD individuals with normal AD biomarkers (A−T−N−) (A), analysis of individual AT(N) subgroups: A+T+N+, A+T+N−, A+T−N−, A−T+N+, A−T+N− and A−T−N− (B). Data were statistically analysed by Analysis of Variance (ANOVA) followed by Bonferroni/Dunn post hoc test. *, *p* ≤ 0.05, **, *p* ≤ 0.01 and ***, *p* ≤ 0.001. Data are presented as box plots generated using StatView software.

Considering the central role of Aβ in AD pathology, our next step was to investigate whether there is an association between Aβ and specific LFP. To accomplish this, we used the AT(N) profile (Table 2) [26]. By dividing subjects according to this classification, we could monitor changes in LFP depending on the presence of individual pathological markers of AD. First, we compared the levels of specific LFP at the identified fluorescence maxima (290/350 nm, 333/358 nm, 285/335 nm, 310/360 nm and 350/400 nm; excitation/emission) among the AD continuum group, the group of non‐AD individuals with pathological changes, and the group of non‐AD individuals with normal biomarkers. We confirmed statistically significantly elevated levels of LFP in the AD continuum group compared to the group of non‐AD individuals with pathological changes at two fluorescence maxima, 290/350 nm (*p* = 0.003) and 310/360 nm (*p* = 0.011). Similarly, significantly higher levels were observed in the AD continuum group compared to the group of non‐AD individuals with normal biomarkers (290/350 nm, *p* = 0.007 and 310/360 nm, *p* = 0.004). The results for the 310/360 nm fluorescence maximum are shown in (Figure 4A). Increased fluorescence intensity was also detected in the AD continuum group compared to the group of non‐AD individuals with pathological changes at the 285/335 nm fluorescence maximum, although with a lower statistical power (0.822).

Furthermore, we stratified both the AD continuum group and the group of non‐AD individuals with pathological changes into subgroups based on the positivity and negativity of two other biomarkers, T/p‐tau and N/neurodegeneration (Table 2). The LFP levels in subgroups with positive Aβ status did not differ significantly from each other but were elevated compared to subgroups with negative Aβ status. This trend was observed across three fluorescence maxima (290/350 nm, 285/335 nm and 310/360 nm). Statistically significant differences were confirmed at two fluorescence maxima, 285/335 nm (*p* = 0.003) and 310/360 nm (*p* = 0.003) for A+T+N+ versus A−T−N−; however, with a lower statistical power (285/335 nm = 0.884 and 310/360 nm = 0.831). The results for the 310/360 nm fluorescence maximum are presented in (Figure 4B). Additionally, LFP levels in Aβ‐negative subgroups, within the group of non‐AD individuals with pathological changes (A−T+N+, A−T+N−), were low and comparable to the group of non‐AD subjects with normal biomarkers (A−T−N−) (Figure 4B).

### 3.3 Correlation Analyses Between CSF Biomarkers and LFP in Blood

The concentrations of CSF biomarkers (Aβ42, p‐tau181 and t‐tau) are used to confirm AD diagnosis in clinical practice. Therefore, we investigated whether these biomarkers correlate with specific LFP in the blood. Pearson's correlation analysis revealed a moderate negative correlation between erythrocyte LFP levels and CSF Aβ42 concentrations at three significant fluorescence maxima identified in the quantitative analysis: 290/350 nm (*r* = −0.488, *p* = 0.004), 285/335 nm (*r* = −0.566, *p* < 0.001) and 310/350 nm (*r* = −0.542, *p* = 0.002). Additionally, a moderate positive correlation was observed between erythrocyte LFP levels and CSF p‐tau181 concentrations, specifically at 285/335 nm (*r* = 0.427, *p* = 0.011) and 310/350 nm (*r* = 0.477, *p* = 0.007). In contrast, no significant or only weak positive correlations were found between erythrocyte LFP levels and CSF t‐tau concentrations. To evaluate whether the observed correlations were independent of age and sex, we performed multiple linear regression analyses for the significant fluorescence maxima. The variance of Aβ42 in relation to LFP levels was as follows: 290/350 nm (∆R^2^ = 0.249, *p*_*Aβ42*_ = 0.004), 285/335 nm (∆R^2^ = 0.314, *p*_*Aβ42*_ < 0.001), 310/360 nm (∆R^2^ = 0.276, *p*_*Aβ42*_ = 0.003); and for p‐tau181, the association was: 285/335 nm (∆R^2^ = 0.153, *p*_*p‐tau181*_ = 0.022) and 310/360 nm (∆R^2^ = 0.198, *p*_*p‐tau181*_ = 0.013). The results confirmed statistically significant associations between erythrocyte LFP levels and CSF biomarker concentrations (Aβ42, p‐tau181), independent of age and sex.

## 4 Discussion

In our work, we confirmed increased LFP levels in erythrocytes of participants with AD. Significantly higher levels of LFP compared to CU were detected in the MCI stage for fluorescence maxima at 290/350 nm, 333/358 nm, 285/335 nm and in the dementia stage at 290/350 nm, 333/358 nm, 285/335 nm and 310/360 nm (excitation/emission). In our previous study we found significantly higher levels of LFP in erythrocytes in AD compared to controls in the MCI stage at 290/350 nm, 333/358 nm, 285/335 nm, 310/360 nm and 350/400 nm and in the dementia stage at 285/335 nm. On top of that, there were significantly higher levels of LFP in MCI versus dementia stage at the 310/360 nm fluorescence maximum (excitation/emission) [13]. Despite some small differences in LFP between the previous and current study that might result from improving diagnosis of AD aetiology in the current study and including biomarker‐defined subjects, the data are consistent and confirm increased LFP levels in erythrocytes of participants with AD, including MCI stage. We have repeatedly shown that specific LFP are elevated in blood in AD and can be detected by fluorescence spectroscopy in early stages of the disease.

OS is considered one of the key factors in the pathogenesis not only of AD but also of other neurodegenerative disorders, including LBD, FTLD, amyotrophic lateral sclerosis or Parkinson's disease [14, 15, 16, 30, 31]. Despite the presence of OS in non‐AD aetiologies, our results demonstrate that the analysed LFP in erythrocytes were specific to AD and not elevated in non‐AD pathology (FTLD/LBD). We hypothesise that this is because LFP are not general markers of OS (such as malondialdehyde, 4‐hydroxynonenal, protein carbonyls) but rather represent unique compounds. Their molecular composition is likely disease‐specific, with the LFP profile in AD differing from that associated with non‐AD conditions. This hypothesis is supported by the fact that three fluorescence maxima identified in AD‐MCI (290/350 nm, 285/335 nm and 310/360 nm, excitation/emission) were significantly increased compared to non‐AD MCI. Moreover, the same three fluorescence maxima were also elevated in ADD compared to non‐AD MCI. These fluorescence maxima thus appear to be specific for AD‐related pathology at the early stage and may potentially serve as specific peripheral markers.

Important insights into the pathogenesis of AD and the relationship between Aβ and specific LFP can be obtained by analysing LFP levels in groups classified according to the AT(N) framework. In this system, A+/− indicates the presence or absence of amyloid pathology, T+/− refers to tau pathology and N+/− reflects neurodegeneration. Individuals who are negative for all three biomarkers show no evidence of amyloid plaques, tau pathology, or neurodegeneration [26, 27, 28, 29]. In our study, we confirmed a significantly increased amount of LFP in individuals who were A+ (indicating amyloid pathology) compared to A− individuals for the fluorescence maxima at 290/350 nm and 310/360 nm (excitation/emission). Multiple mechanisms linking Aβ to OS have been described [7]. Aβ disrupts normal mitochondrial function, leading to primary OS, impaired ATP production and overall metabolic disruption [32]. Neurons, which have high energy demands for processes such as action potential generation, signal transduction and axonal transport, are particularly vulnerable. Aβ reduces the efficiency of electron transfer in the electron transport chain, primarily at complexes I and III. This dysfunction increases the production of reactive oxygen species, resulting in damage to mitochondrial macromolecules [33]. Furthermore, Aβ inhibits mitochondrial superoxide dismutase, a key enzyme that detoxifies superoxide radicals, thereby exacerbating oxidative damage [34]. Aβ also binds and inhibits Aβ‐binding alcohol dehydrogenase, an enzyme normally involved in the detoxification of harmful aldehydes [35]. This inhibition promotes lipid peroxidation, enhances the production of reactive oxygen species and leads to further mitochondrial damage.

On the other hand, OS can promote increased production and toxicity of Aβ. Free radicals have been shown to upregulate amyloid precursor protein expression, leading to elevated intracellular and extracellular Aβ levels [36]. Moreover, OS has been associated with increased activity of β‐secretase 1 [37], as well as γ‐secretase. Notably, the OS‐induced upregulation of β‐secretase 1 is regulated via γ‐secretase–dependent mechanisms [38, 39]. Although the causal relationship between Aβ accumulation and OS in the development of AD remains unclear, our findings suggest a possible association between Aβ pathology and the generation of specific LFP. Within the Aβ‐positive group, we defined subgroups based on tau pathology and neurodegeneration status (A+T−N−, A+T+N− and A+T+N+), which are thought to reflect different stages along the AD continuum. LFP levels were significantly higher in the A+T+N+ group compared to individuals with normal AD biomarkers (A−T−N−). In the A+T−N− and A+T+N− subgroups, a similar trend was also observed. Moreover, LFP levels did not differ significantly among the individual A+ subgroups, meaning that regardless of whether tau pathology or neurodegeneration is present, specific LFP are probably produced in the presence of amyloid pathology. These findings are consistent with the hypothesis that Aβ is a key contributor to oxidative processes involved in the production of specific LFP [40].

In contrast to AD, Aβ does not play a central role in the pathogenesis of primary tauopathies, such as FTLD [16]. As a result, pathological processes associated with Aβ production, aggregation and the formation of specific LFP are not activated. This is supported by our findings, where LFP levels in amyloid‐negative (A−) but tau‐positive (T+) individuals were low and comparable to those in biomarker‐negative individuals. This does not imply that LFP are entirely absent in non‐AD conditions, but rather that their composition likely differs, exhibiting distinct fluorescence maxima. Although AD is also characterised by tau pathology, it occurs downstream of Aβ accumulation [41]. This differentiates AD from primary tauopathies, in which OS arises through alternative mechanisms, and the disease‐specific LFP detected in AD is not present.

In this study, we investigated the potential relationship between erythrocyte‐derived LFP and CSF biomarkers, specifically Aβ42, p‐tau181 and t‐tau. Our findings demonstrate moderate yet statistically significant correlations between erythrocyte LFP levels and key CSF biomarkers, suggesting that erythrocyte LFP may reflect central neurodegenerative changes. We observed consistent moderate negative correlations between LFP fluorescence intensities and CSF Aβ42 concentrations at three fluorescence maxima (290/350 nm, 285/335 nm and 310/350 nm). These findings indicate that increased levels of LFP in erythrocytes are associated with lower concentrations of CSF Aβ42, a hallmark of AD pathology. Furthermore, moderate positive correlations were observed between LFP levels and CSF p‐tau181 at the 285/335 nm and 310/350 nm fluorescence maximum. Notably, no significant associations were found between LFP levels and t‐tau, potentially indicating that LFP are more reflective of phosphorylation‐specific tau changes rather than general neuronal damage. To account for possible confounding effects of age and sex, multiple linear regression analyses were performed. The models confirmed that associations between LFP levels and CSF Aβ42 and p‐tau181 remained statistically significant even after adjustment. The explained variance (ΔR^2^) ranged from ~25% to 31% for Aβ42 and from ~15 to 20% for p‐tau181, highlighting the multifactorial nature of AD, where no single biomarker is likely to show a one‐to‐one correspondence with disease mechanisms [42, 43, 44]. Overall, our data support the hypothesis that erythrocyte LFP are associated with established CSF biomarkers of AD.

This study has several limitations that should be acknowledged. OS is a shared pathogenic mechanism in various neurodegenerative disorders. Even though we included a limited size group of non‐AD subjects to confirm the specificity of LFP for AD aetiology and evaluate their potential as AD‐specific biomarker, future research should include other non‐AD pathologies associated with OS, such as amyotrophic lateral sclerosis, Parkinson's disease and others. Moreover, the observed correlations between LFP levels and CSF biomarkers exhibited only modest explained variance. Therefore, future studies should include larger and more diverse cohorts to better assess the clinical utility of LFP. Finally, the chemical characterisation of LFP using high‐performance liquid chromatography or mass spectrometry is necessary to gain deeper insight into its molecular composition. This may enhance our understanding of AD pathogenesis and support the development of targeted therapeutic strategies.

In conclusion, we demonstrated the presence of specific LFP in erythrocytes of biomarker‐defined individuals with AD, detectable even in the early stages of the disease. These specific LFP appear to be generated in response to Aβ overproduction and are absent in other neurodegenerative conditions. Importantly, LFP levels in erythrocytes correlate with established CSF biomarkers and may serve as a promising AD‐specific, blood‐based biomarker that reflects pathophysiological processes in the brain.

## Author Contributions

**Lenka Martináková:** conceptualization, methodology, formal analysis, investigation, data curation, writing – original draft, visualization, project administration, funding acquisition. **Zuzana Chmátalová:** conceptualization, validation, writing – original draft. **Kateřina Veverová:** methodology, resources, data curation, writing – review and editing. **Vanesa Jurášová:** investigation, data curation. **Alžběta Katonová:** investigation, data curation. **Martina Laczó:** investigation, data curation. **Jan Laczó:** validation, investigation, writing – review and editing. **Martin Vyhnálek:** investigation, writing – review and editing, funding acquisition. **Jakub Hort:** investigation, writing – review and editing, funding acquisition. **Alice Skoumalová:** conceptualization, writing – original draft, supervision, project administration, funding acquisition, validation.

## Funding

This work was supported by Grantová Agentura, Univerzita Karlova (180221), NextGenerationEU (LX22NPO5107), Ministerstvo zdravotnictví Ceské Republiky (00064203).

## Conflicts of Interest

The authors declare no conflicts of interest.

## Data Availability

The data that support the findings of this study are available from the corresponding author upon reasonable request.
